# High-intensity focused ultrasound (HIFU) for the treatment of female urinary incontinence: A retrospective analysis

**DOI:** 10.1097/MD.0000000000039940

**Published:** 2024-10-04

**Authors:** Aylin Önder Dirican, Mehmet Ufuk Ceran, Oğuzhan Kahraman, Mehmet Giray Sönmez

**Affiliations:** a Department of Gynecology and Obstetrics, Faculty of Medicine, Baskent University, Konya, Turkey; b Department of Urology, Faculty of Medicine, Baskent University, Konya, Turkey; c Department of Urology, Faculty of Medicine, Necmettin Erbakan University, Konya, Turkey.

**Keywords:** high-intensity focused ultrasound, quality of life, urinary incontinence

## Abstract

This study aims to demonstrate the effectiveness of high-intensity focused ultrasound, a noninvasive treatment, for managing urinary incontinence (UI) in women. This is a single-center, retrospective study involving 28 women. Patients, aged between 32 and 65, were included. Patients with insulin-dependent diabetes, neurological disease, active urinary tract infection, undiagnosed vaginal bleeding, who had incontinence surgery, and receiving estrogen therapy were excluded from the study. Incontinence severity was evaluated with the International Incontinence Consultation Questionnaire Short Form (ICIQ-SF). Patients were evaluated before treatment and 6 months after treatment using the ICIQ-SF and the Pelvic Organ Prolapse/Urinary Incontinence Sexual Function Assessment short form. In the analysis of numerical variables independent or paired *t* test or linear mixed effects models were used. Least square means were used in post hoc comparisons. Mean age of the patients was 45.50 ± 7.59 years. There were 18 (64%) stress urinary incontinence (SUI) and 10 (36%) mixed urinary incontinence (MUI). Six months after treatment, mean ICIQ-SF and Pelvic Organ Prolapse/Urinary Incontinence Short Form Questionnaire scores showed a significant positive change. After the procedure, UI completely disappeared in 43% of the patients. The rate of severe UI decreased from 39% to 8%, and very severe UI decreased from 8% to 0%. Incontinence severity was significantly different in the MUI and SUI groups before and after the procedure. After the procedure, UI completely disappeared in 67% of the patients in the SUI group, while it remained at a mild level in 33%. The decrease in ICIQ-SF score in the SUI group was significantly higher than that in the MUI group. There were no severe adverse events, in 4 patients there was mild vaginal discharge which resolved in 1 week. This study showed that high-intensity focused ultrasound treatment, can be effective and safe even in a single session. Selection and recall biases are potential biases in retrospective studies. Lacking a control group is another limitation. Although advances in technology are very important for medical treatments, their effectiveness and safety need to be proven. Future research in this area with a larger sample size and a prospective design will offer further evidence supporting effectiveness of this treatment model.

## 1. Introduction

Urinary incontinence (UI) is a condition that negatively affects people’s quality of life and affects approximately 50% of women.^[1]^ There are 2 main types resulting from different pathophysiological mechanisms and different etiological factors: stress urinary incontinence (SUI) and urgency urinary incontinence. Mixed urinary incontinence (MUI) is the co-occurrence of both types.^[2]^

SUI is defined as UI that occurs with increased intra-abdominal pressure, such as coughing, sneezing, or strenuous exercise. Although there is no clear consensus, conditions such as advancing age, genetics, pregnancy, obesity, pelvic trauma, constipation, and chronic disease may increase the risk of UI in women by causing changes in the pelvic floor structure.^[3]^ There are technically 2 different etiologies for SUI: intrinsic sphincter deficiency and urethral hypermobility due to poor fascial/ligamentous support of the urethra and bladder neck. The purpose of treatment is the strengthening of pelvic floor support and the restoration of normal sphincter function.^[4]^

There are nonsurgical treatments (e.g., lifestyle changes, Kegel exercises, biofeedback, and pelvic floor stimulation) that are recommended as the first line and are effective in strengthening pelvic floor structures.

However, difficulties and compliance problems may occur when continuing the treatment for a long time.^[5,6]^

Various surgical options include traditional Burch colposuspension, bladder neck slings, and modifications thereof. The most commonly used surgical techniques in the treatment of SUI are retropubic and transobturator mid-urethral sling methods, which are based on supporting the mid-urethra with a synthetic patch and are considered minimally invasive.^[7]^ Although these techniques are highly effective, there are concerns about their long-term risks. On the other hand, current surgical treatments may be less preferred because they require anesthesia, incision, and may affect future pregnancies.^[8,9]^ Advances in technology can provide good clinical outcomes and short recovery times, perhaps with less invasive methods.^[10]^

In recent years, energy-based options such as radiofrequency (RF) and laser, which are considered to be noninvasive and safe, have attracted attention in pelvic floor problems and genital tissue regeneration.^[11,12]^ Especially in the literature, there is histological and clinical evidence for energy-based treatments in genital tissue regeneration.^[13–17]^ Studies on vaginal thermal treatments have mostly focused on the functional renewal of the vaginal mucosa. However, in deeper pelvic floor problems such as UI, it may be important to evaluate the effect on submucosal tissues. CO_2_ and erbium lasers are not effective in submucosal regions considering their wavelengths.^[11,13,14]^ RF provides a deeper effect than lasers, but its theoretical thermal effect at sufficient depth is still debated.^[12,17]^ In July 2018, Food and Drug Administration issued an alert stating that more studies are needed to prove their effectiveness and safety.^[18]^

Focused ultrasound therapy, a noninvasive treatment method, appears as a promising approach in many clinical applications. High-intensity focused ultrasound (HIFU) focuses high-energy focal acoustic light on the desired tissues, creating a thermal effect on the target tissue without damaging the upper and surrounding tissues. Through focal thermal damage points, it facilitates the reorganization of collagen within a specified depth layer using heat that can exceed 65 °C for 0.1 seconds. Success of focused ultrasound depends on accurate targeting and controlled and sufficient energy deposition in the target volume.^[19]^ Today, HIFU is used in a wide variety of areas, from dermatological diseases to the treatment of benign or malignant tumors.^[20]^ Many studies have shown that HIFU can be safe and effective in gynecological diseases such as leiomyoma and adenomyosis.^[21,22]^

It is important to note that HIFU, primarily used for solid tumor ablation, utilizes a high-energy ultrasound beam to create ablation through a thermo-mechanical effect.^[23]^ In contrast, HIFU devices designed for dermatological aesthetic treatments, vaginal rejuvenation, and UI operate with lower energy parameters (0.4–1.2 J/mm², 4–10 MHz, and 1.5–4.5 mm focal depth), resulting in a purely thermal effect rather than an ablative one.^[24]^

Unfortunately, there is insufficient evidence in the literature regarding the use of HIFU, which is widely marketed for vaginal rejuvenation and UI treatment. We found only one study evaluating the effects of HIFU on UI in women.^[25]^ Considering the relatively invasive nature of existing treatment modalities, the limited number of studies on the safety and efficacy of the noninvasive modality HIFU for treating UI in women is striking.

It is a collective responsibility to further investigate thermally effective vaginal treatment models to ensure their safe implementation. This study was a retrospective analysis aimed at evaluating the effectiveness and safety of HIFU in treating female urinary incontinence. A total of 28 patients who underwent HIFU treatment were included in the study. Patient data were collected and reviewed retrospectively to assess treatment outcomes and any associated adverse effects. This design enables a comprehensive assessment of HIFU’s impact on UI in a real-world clinical setting.

## 2. Materials and methods

This single-center, retrospective study was conducted between November 2021 and December 2023 at the Gynecology and Obstetrics Clinic of a university hospital. The study was planned in accordance with the 2013 Declaration of Helsinki of the World Medical Association and local ethics committee approval was obtained for the study (Karatay University Medical Research Ethics Committee). All patients were informed about the procedure beforehand their informed consent was obtained.

### 2.1. Study population

A total of 28 women aged 30 to 65, sexually active and diagnosed with SUI were included in the study. All patients who met the inclusion and exclusion criteria and whose data were available were included in the study. Specifically, all eligible patients based on the predefined criteria were included, ensuring that our sample accurately represents the population under investigation. Due to the retrospective nature of the study, no predetermined sample size was set prior to data collection.

### 2.2. Inclusion criteria

Patients, who had a negative PAP smear (Papanicolau cytology) test in the previous 6 months, were not pregnant, and had no uterine or adnexal mass detected on pelvic ultrasonography were included in the study.

### 2.3. Exclusion criteria

Patients with insulin-dependent diabetes, neurological disease, active urinary tract infection, undiagnosed vaginal bleeding, those who had undergone surgery for incontinence, and those receiving estrogen therapy were excluded from the study. Additionally, women with a cystocele degree above 2 according to the POP-Q classification were not included.

### 2.4. Data collection

Demographic and clinical characteristics of the patients were examined through the hospital data system. During the clinical evaluation, the patients’ anamnesis, physical and pelvic examination, urinalysis, urine culture, and provocative stress test results were recorded. Age, gravidity, parity, type of birth, body mass index (BMI), and menopause status were recorded as demographic characteristics. BMI was calculated based on height and weight (expressed in kg/m^2^).

### 2.5. Diagnosis of urinary incontinence

Stress UI and MUI were diagnosed by history, pelvic examination, and provocative stress test. A provocative stress test was performed on each patient while standing, after 500 mL oral hydration. None of the patients with MUI whose SUI symptoms were more severe were using medication. Patients considered to have SUI are those with more severe SUI symptoms.

The severity of UI and its impact on quality of life was evaluated by a nurse by interviewing each patient individually and using the International Consultation Incontinence Questionnaire-Short Form (ICIQ-SF).^[26]^ A Turkish adaptation of this questionnaire was available.^[27]^ According to ICIQ-SF results, UI is divided into 4 grades: no UI (0), mild (1–5), moderate (6–12), severe (13–18), and very severe (19–21).^[28]^

Additionally, to evaluate the effect of UI on sexual function, the Pelvic Organ Prolapse/Urinary Incontinence Short Form Questionnaire (PISQ-12), which was previously validated in Turkish, was used. PISQ-12 short form is a validated test that evaluates female sexual function.^[29,30]^ Patients were asked to fill out the questionnaire both before the procedure and at the examinations 6 months after the procedure.

The improvement in UI symptoms and their impact on quality of life and sexual function were evaluated using ICIQ-SF and PISQ-12 scale scores before and after treatment. Decreased scores on the ICIQ-SF scale were considered symptomatic improvement.

### 2.6. HIFU procedure

In the treatment of all patients, a European Economic Area (CE) certified Uzer brand HIFU ultrasound therapy device was used (HIFU CHINA-2019). The technical specifications of the HIFU device used were as follows: a 4 to 7 MHz frequency range, a 1 to 10 mm focus range, a 0.5 to 25 mm focus treatment line length (1 mm/step), a 0.2 to 2.0 J (0.1 J/step) output power and a 5 to 25° (1°/step) step)angle range. The device contains vaginal transducers with a depth of 3.0 mm and 4.5 mm.

The treatment protocol was planned in 2 steps, taking approximately 20 minutes in total. In the first step, a 3.0 mm (4 MHz) vaginal transducer was used with parameters set to 1.3 J energy, 1.4 mm focal range, and a 25 mm focal treatment line length. Five degree was selected as the angle range and a 360° scan was performed using the automatic scanning mode, starting from 12 o’clock and proceeding clockwise, with 71 shots. In the second step, a 4.5 mm (4 MHz) vaginal transducer was used, and the parameters were set to 1.3 J energy, 1.4 mm focal range, and a 25 mm focal treatment line length. In this step, the angle range was selected as 3°. The anterior 120° area of the 360° vaginal canal, which includes the paraurethral area, was scanned with 40 shots. The scanning started at the 10 o’clock position and proceeded clockwise to the 2 o’clock position, using the automatic scanning mode. The procedure is performed in outpatient clinic conditions, without the need for any preliminary preparation or anesthesia. Additionally, there is no need to use medication after the procedure. After the procedure, patients were discharged with the recommendation to abstain from sexual intercourse for at least 3 days.

The procedure was completed in a single session. All patients were followed up by the same gynecologist 1 month and 6 months after the procedure. The effectiveness of the procedure was evaluated at the 6-month follow-up by provocative stress test, vaginal examination, and refilling of the questionnaires filled out before the procedure.

Eligible women participating in the study completed questionnaires both before treatment and 1 and 6 months after treatment. The primary outcome was a change from the baseline in ICIQ-SF 1 to 6 months after the procedure. The secondary outcome was to compare changes in sexual function on the PISQ-12 to the baseline in women with UI 1 to 6 months after the procedure.

### 2.7. Statistical analysis

For numerical variables, mean, standard deviation, frequency, and percentage statistics are given for categorical variables. Statistical analyses were performed using R version 4.3.2 (R Core Team, 2024), with significance set at *P* < .05. In the analysis of numerical variables independent or paired *t* test or linear mixed effects models were used. Least square means were used in post hoc comparisons. Given that the proportion of missing data in present study is small, complete case analysis was used. This method involves excluding cases with missing data and will be applied cautiously to ensure that the amount of missing data remains minimal and does not introduce significant bias into the results. This approach was only used if the proportion of missing data is low and its exclusion does not significantly impact the overall analysis. Analyzes were made with the R 4.3.2 (R Core Team, 2024) program, and *P* < .05 was considered significant.

## 3. Results

The general demographic and clinical characteristics of the patients are shown in Table 1.

**Table 1:** Characteristics of the patients before the intervention.

	n = 28*
Age (years)	45.50 ± 7.59
Parity	2.43 ± 0.79
Delivery type	
Cesarean only	8 (29)
Vaginal	20 (71)
BMI (kg/m^2^)	24.09 ± 1.66
*Menopausal status*	
Premenopausal	21 (75)
Post-menopausal	7 (25)
*Incontinence type*	
MUI	10 (36)
SUI	18 (64)
ICIQ-SF score	12.32 ± 4.87
*ICIQ-SF grade*	
No UI	0 (0)
Mild	4 (14)
Modarate	11 (39)
Severe	11 (39)
Very severe	2 (8)
PISQ-12 score	28.36 ± 6.53

BMI = body mass index, ICIQ-SF = International Consultation Incontinence Questionnaire-Short Form, MUI = mixed urinary incontinence, PISQ-12 = Pelvic Organ Prolapse/Urinary Incontinence Sexual Questionnaire, SUI = stress urinary incontinence, UI = urinary incontinence.

* Mean±SD n (%).

After the procedure, the mean ICIQ-SF score decreased significantly (*P* < .001). Mean PISQ-12 score increased significantly (*P* < .001) after the procedure (Table 2).

**Table 2:** ICIQ-SF and PISQ scores before and 6 months after HIFU procedure.

	Total (n = 28)*		*P*-value†
	Before	After	
ICIQ-SF score	12.32 ± 4.87	4.54 ± 5.30	<.001
PİSQ-12 score	28.36 ± 6.53	35.86 ± 6.30	<.001

* Mean ± SD.

† Paired *t* test.

UI completely disappeared in 42% of the patients. Additionally, the rate of severe UI decreased from 39% (n = 11) to 8% (n = 2), and the rate of very severe UI was reduced to zero (Table 3).

**Table 3:** ICIQ-SF grades before and after the intervention.

After					
	No UI, 12 (42)	Mild, 7 (25)	Moderate, 7 (25)	Severe, 2 (8)	Very severe, 0 (0)
Before					
No UI	0 (0)	0 (0)	0 (0)	0 (0)	0 (0)
Mild	4 (33)	0 (0)	0 (0)	0 (0)	0 (0)
Moderate	7 (58)	4 (57)	0 (0)	0 (0)	0 (0)
Severe	1 (8.3)	3 (43)	6 (86)	1 (50)	0 (0)
Very severe	0 (0)	0 (0)	1 (14)	1 (50)	0 (0)

n (%), UI = urinary incontinence.

When the variables were compared in terms of SUI and MUI types; mean age, parity, BMI, and menopause status were similar in both groups (*P* = .35, *P* = .42, *P* = .10, *P* = .063, respectively).

After the procedure, UI completely disappeared in 67% of the patients in the SUI group and remained at a mild level in 33%. In the MUI group, although the severity of UI generally decreased, it did not completely disappear (Table 4).

**Table 4:** Before and after comparison according to incontinence type.

	MUI, n = 10*	SUI, n = 18*	*P*-value†
Age(years)	47.40 ± 8.07	44.44 ± 7.33	.35
Parity	2.60 ± 0.84	2.67 ± 1.28	.74
*Delivery type*			>.99
Cesarean only	3 (30)	5 (28)	
Vaginal	7 (70)	13 (72)	
BMI (kg/m^2^)	24.83 ± 1.80	23.68 ± 1.48	.10
*Menopausal status*			.063
Premenopausal	5 (50)	16 (89)	
Post-menopausal	5 (50)	2 (11)	
ICIQ-SF score before	16.90 ± 1.85	9.78 ± 4.08	<.001
PISQ-12 score before	23.70 ± 4.76	30.94 ± 5.99	.002
ICIQ-SF score after	10.90 ± 3.18	1.00 ± 1.50	<.001
PISQ-12 score after	31.00 ± 5.54	38.56 ± 5.02	.002

BMI = body mass index, ICIQ-SF = International Consultation Incontinence Questionnaire-Short Form, MUI = mixed urinary incontinence, PISQ-12 = Pelvic Organ Prolapse/Urinary Incontinence Sexual Questionnaire, SUI = stress urinary incontinence.

* Mean±SD; n (%).

† Welch Two Sample *t* test; Fisher’s exact test.

**Figure 1.**
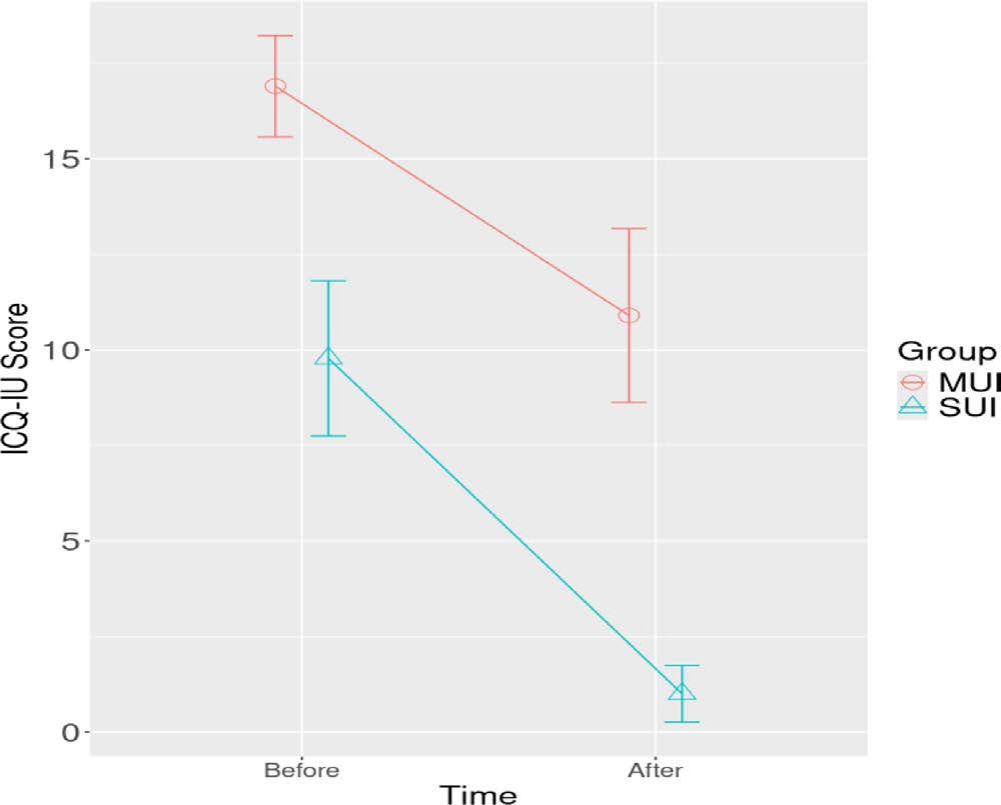
The change in ICIQ-SF scores with respect to time, group, and time–group interactions was examined, and it was found that the decrease in scores was greater in the SUI group compared to the MUI group. ICIQ-SF = International Consultation Incontinence Questionnaire-Short Form, MUI = mixed urinary incontinence, SUI = stress urinary incontinence.

In the mixed models, the changes in ICIQ-SF and PISQ-12 scores of MUI and SUI over time were investigated. In the mixed model for ICIQ-SF score, time, group, and time-group interaction effects were found to be significant (*P* < .001, *P* < .001, and *P* = .036, respectively). Decrease in ICIQ-SF score in the SUI was more significant than in the MUI group (Table 5 and Fig. 1).

**Table 5:** Temporal interaction of incontinence subgroups according to ICIQ-SF score.

Contrast	Group	Estimate	SE	df	t-ratio	*P*-value
Before–after	MUI	6.000	1.010	26.000	5.942	.000
Before–after	SUI	8.778	0.753	26.000	11.663	.000

MUI = mixed urinary incontinence, SE = standard error, SUI = stress urinary incontinence.

**Figure 2.**
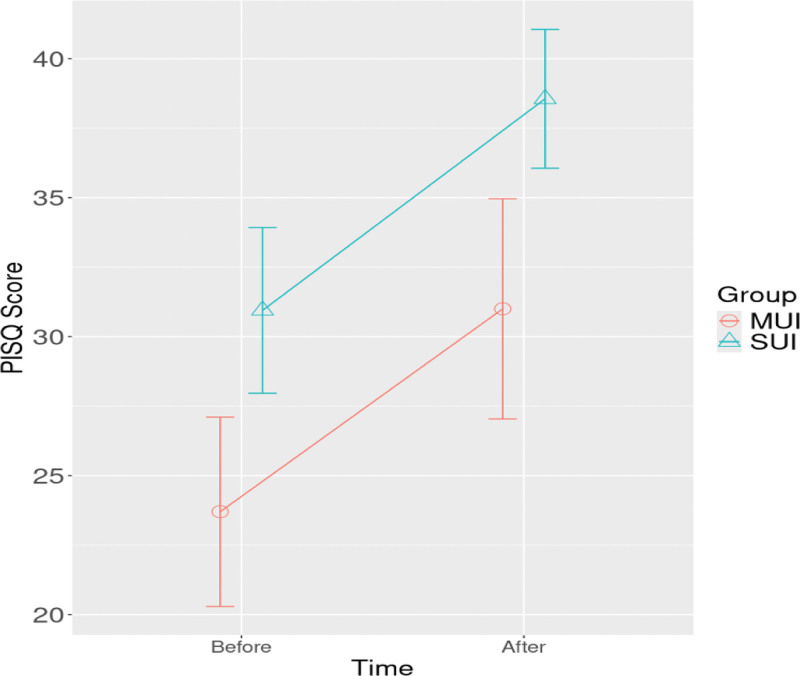
In the analysis of the PISQ-12 score, increases were observed over time, as well as in group and time–group interactions, for both the MUI and SUI groups. PISQ-12 = Pelvic Organ Prolapse/Urinary Incontinence Sexual Questionnaire, MUI = mixed urinary incontinence, SUI = stress urinary incontinence.

In the mixed model for PISQ-12 score, while time and group effects were found to be significant (*P* < .001, *P* = .001), the interaction effect was not significant (*P* = .82). The increase in PISQ-12 scores were not significant between the groups (Table 6 and Fig. 2).

**Table 6:** Temporal interaction of incontinence subgroups according to PISQ-12 score.

Contrast	Group	Estimate	SE	df	t-ratio	*P*-value
Before–after	MUI	‐7.300	1.082	26.000	‐6.747	.000
Before–after	SUI	‐7.611	0.806	26.000	‐9.438	.000

MUI = mixed urinary incontinence, SE = standard error, SUI = stress urinary incontinence.

HIFU treatment was painless and well tolerated for all the patients. Immediately following HIFU, there was no visible swelling or lesions on the vaginal mucosa other than mild redness in all the patients. In the first week follow-up, the patients were questioned about their discomfort such as burning, stinging, and vaginal discharge. Mild vaginal discharge was observed in only 4 patients, and the symptom disappeared within 1 week without requiring any treatment.

## 4. Discussion

This study is one of very few studies evaluating the effects of noninvasive and non-ablative HIFU in the treatment of UI. Our results showed that HIFU can be effective in the treatment of UI, especially in a single session, without causing serious side effects. Although its effectiveness was found to be particularly high in women with SUI, it reduced the severity of symptoms in women with MIU. Additionally, we observed that it may positively impact the sexual function of the participants as a secondary effect.

Loss of support from specialized tissues such as pelvic floor muscle, connective tissue, and fascia disrupts the stability of the urethra and bladder neck.^[2]^ SUI treatment is provided by supporting the pelvic floor tissues with conservative approaches or surgically.^[4]^ Less invasive methods are now preferred in SUI surgery. However, complications such as loss of sexual function, chronic pain, infection, difficulty urinating, recurrent urinary incontinence, permanent nerve damage, or damage to surrounding organs may occur due to the use of a pelvic mesh.^[8]^

For many women with SUI, mesh surgery is a viable option and is highly effective.^[7]^ However, this may not be true for everyone, and the important thing is to enable these women to make fully informed decisions based on clear information about benefits, risks, and alternatives.^[9]^ In this sense, participants in current the study made their decisions after evaluating all treatment options for SUI.

In recent years, thermal energy-based treatments for UI, genitourinary syndrome, and vaginal looseness have come to the fore.^[11,12,31]^ Thermal therapy may be a noninvasive alternative treatment option, especially for SUI. Moderate thermal exposure to pelvic support tissues will alter the collagen in target tissues, and tissue damage caused by neocollagenesis will cause tightening and remodeling of the target tissue.^[32]^ The aim of thermal therapy, especially in SUI treatment, should be to reshape and tighten the vaginal submucosal tissues and endopelvic fascia without damaging adjacent tissue layers (e.g., urethra or vaginal wall mucosa).^[33]^ Although laser therapy for SUI has been found effective in many studies, unwanted thermal damage to the vaginal mucosa may occur due to stronger absorption of photons in the urethral and vaginal mucosa than in deeper tissues. In this sense, considering the limitations of other thermal techniques in the treatment of SUI, HIFU may be more effective with targeted energy penetration at the desired depth and may be advantageous in protecting nontarget areas.^[11,15]^

Historically, ultrasound in medicine was used first for therapeutic purposes and then for diagnostic purposes. Ultrasound frequency ranges used in diagnosis and treatment are different. Therapeutic applications require higher power at lower frequencies (1 MHz).^[33]^ The use of ultrasonic energy in treatment is based on the principle that if the temperature in any focused target tissue rises above 70 °C in <1 second, it causes coagulation and necrosis in the focal tissue.^[34]^ There are studies demonstrating its use in various gynecological pathologies, primarily due to its thermal-ablative effects. It is a treatment method with proven safety and effective therapeutic outcomes, particularly in symptomatic uterine myomas. Liu X. et al, in their study on HIFU ablation therapy for women with uterine fibroids, showed significant improvement in symptoms and quality of life, along with a low long-term reintervention rate.^[35]^ In other studies, its use has also been shown to have a low side effect profile in the treatment of cervical lesions, vulvar dystrophies, uterine adenomyosis, abdominal wall endometriosis, and pregnancies with cesarean scars.^[20,22,36,37,38]^ Although HIFU treatment for benign diseases in the womb is generally considered safe, early skin burns and nerve injuries have also been reported.^[39]^ However, it has been shown that the rate of side effects has decreased significantly with the development of the technique and the increase in the experience of physicians.^[40]^

Low-energy HIFU devices, which have been used in vaginal rejuvenation and UI treatment in recent years, are inspired by dermatological aesthetic devices. They are specifically manufactured to precisely deliver the thermal effect to the targeted area, at a greater treatment depth than that offered by RF and laser techniques.^[14,25]^ Taking into account the vaginal anatomy, the thicknesses of the vaginal wall, endopelvic fascia, and urethral wall are approximately 2.7 mm, 4.3 mm, and 2.4 mm, respectively.^[41]^ The tissue penetration depths for laser and RF devices are 1.5 mm and 3 mm, respectively.^[31]^ A key component of HIFU treatment, the vaginal transducer can direct the ultrasound beam into the submucosal layers of the vagina and even the endopelvic fascia.^[42]^ Therefore, rotating movements of the probe, especially in this region, will increase its effectiveness. In our study, we used a 4.5 mm deep vaginal transducer specific to the paraurethral region. Therefore, our results for SUI were quite successful.

A HIFU device very similar to the one used in this study was used in the study by Elías et al, and the effectiveness of HIFU in the treatment of vaginal atrophy, pelvic organ prolapse, and SUI was demonstrated.^[25]^ In their study, Elias et al applied 2 therapy sessions 30 to 45 days apart, using an additional 4.5 mm deep transducer to SUI patients. They scanned the paraurethral areas bilaterally with a 40° rotation. And ultimately they showed statistically significant clinical improvement for SUI. In our study, the anterior vaginal wall was scanned in only 1 session with a 4.5 mm transducer and 120° rotation angle. Our findings according to ICIQ-SF scores were consistent with this study. There was complete recovery, especially in women with mild, moderate, and severe SUI, and significant relief of symptoms in women with very severe SUI. Additionally, it was observed that the severity of UI was alleviated in women with MUI. A larger retropubic area may allow the placement of more ultrasonic beams and therefore increase the effect of the treatment, whereas exposure in a smaller area may limit the treatment and possibly increase the risk of thermal damage to nontarget tissues.^[33]^ From this angle, the anterior vaginal wall was scanned at a wider angle. Perhaps this may explain the effectiveness of the treatment even in a single session.

Previous studies have shown that ultrasound energy causes an increase in the synthesis of collagen, angiogenesis factors, interleukin-8, basic fibroblast growth factor, and vascular endothelial growth factor in tissues.^[36]^ Again in their study, Elias et al thickening of the vaginal epithelium and lamina propria, better vascularization in the lamina propria, and an increase in estrogen receptor expression in the vaginal wall were observed.^[25]^ In our study, histological evaluation of vaginal tissue was not performed, but the fact that women showed a significant improvement in their sexual lives according to PISQ-12 survey scores may be important in terms of vaginal tissue regeneration. However, it is also a fact that women with UI may avoid sexual intercourse due to fear and embarrassment of UI, especially during sexual intercourse. Therefore, more data is needed to more clearly evaluate the effect of HIFU treatment on the vaginal mucosa.

In our study, the recovery rate in the SUI group was significantly higher than that in the MUI group. This result is consistent with several studies investigating the effect of non-ablative laser treatments in women with UI.^[11,32,42]^ On the other hand, an improvement was also observed in the MUI group, although not completely. This may be due to the effect of MUI on the stress component. In studies where the urgency and stress components of MUI were evaluated separately in laser treatments,^[43]^ it was shown that the urgency component also improved somewhat. The multifactorial nature of UI in terms of pathogenesis can make it difficult for us to predict which patients are most likely to improve. For this reason, especially in MUI, controlled studies should be planned in which urgency and stress components are evaluated separately and factors that may be effective in recovery are also included.

Although the number of cases in the study was low, no significant complications were observed during treatment and subsequent follow-ups. This result is consistent with studies published to date on low-energy HIFU treatments.^[44]^

The perception that tension-free vaginal mesh surgeries (TVT, TOT) are a quick and easy solution for SUI has led to more surgeries and perhaps more complications. Although TVT/TOT are considered minimally invasive surgical procedures, there may be risks of long-term mesh erosion, the possibility of chronic pelvic pain, or situations where it does not meet patients’ expectations. Our short-term results showed that HIFU may be effective and promising in the treatment of SUI without serious side effects. The fact that HIFU is effective in mild, moderate, and severe SUI cases may be important as it offers an alternative that may reduce the need for surgery. On the other hand, HIFU treatment without any additional medical treatment significantly reduced the severity of MUI. In this sense, it can be considered as a supportive alternative to medical treatment in cases of MUI.

## 5. Strength and limitations of the study

The strength of this study is that it is one of the few studies showing the effectiveness of HIFU treatment in women with UI. However, the study had some limitations. The small sample group prevented the effect of the treatment from being evaluated in terms of risk factors, and there was no control group that could make a clearer evaluation. Second, long-term effects, which are important for both the success and safety of treatment, have not been evaluated. However, follow-up of the patients we treat continues and is an important motivation for future studies. The retrospective nature of the present study relies on previously collected data. This can result in incomplete or inconsistent data, leading to potential information bias. Additionally, the reliance on patient records means there could be inaccuracies or omissions in the documentation that affect the study’s findings. Another potential source of bias is selection bias, which can occur because the sample may not represent the broader population due to the specific inclusion and exclusion criteria used. Recall bias is also a concern in retrospective studies, especially when patient self-reports or questionnaires are used to gather data retrospectively. Despite these limitations, we have taken steps to minimize bias wherever possible, such as applying strict criteria for data inclusion and using standardized questionnaires to collect information. Nonetheless, these potential sources of bias should be considered when interpreting the findings.

## 6. Conclusions

This study showed that HIFU treatment, especially in the treatment of SUI, can be effective and safe even in a single session. Although advances in technology are very important for medical treatments, their effectiveness and safety need to be proven. In this sense, controlled studies with larger samples, longer duration, and more variable/predictive factors will provide more evidence of the effectiveness of the presented treatment model.

## Author contributions

**Conceptualization:** Aylin Önder Dirican.

**Data curation:** Mehmet Ufuk Ceran, Mehmet Giray Sönmez.

**Formal analysis:** Aylin Önder Dirican, Mehmet Ufuk Ceran.

**Funding acquisition:** Mehmet Ufuk Ceran.

**Investigation:** Aylin Önder Dirican, Mehmet Giray Sönmez.

**Methodology:** Aylin Önder Dirican.

**Project administration:** Mehmet Ufuk Ceran.

**Resources:** Mehmet Ufuk Ceran.

**Software:** Oğuzhan Kahraman.

**Supervision:** Aylin Önder Dirican, Mehmet Ufuk Ceran, Mehmet Giray Sönmez.

**Validation:** Mehmet Ufuk Ceran.

**Visualization:** Aylin Önder Dirican, Mehmet Ufuk Ceran, Oğuzhan Kahraman.

**Writing – original draft:** Aylin Önder Dirican.

**Writing – review & editing:** Oğuzhan Kahraman.
